# Reply: Reconsidering the interpretation of sperm DNA fragmentation testing in ART decision-making

**DOI:** 10.1093/hropen/hoag046

**Published:** 2026-05-19

**Authors:** Yuanyuan Wang, Rui Wang, Ying Lian, Ben W Mol, Rong Li, Jie Qiao

**Affiliations:** National Clinical Research Centre for Obstetrical and Gynaecological Diseases, State Key Laboratory of Female Fertility Promotion, Centre for Reproductive Medicine, Department of Obstetrics and Gynaecology, Peking University Third Hospital, Beijing, China; NHMRC Clinical Trials Centre, University of Sydney, Sydney, NSW, Australia; National Clinical Research Centre for Obstetrical and Gynaecological Diseases, State Key Laboratory of Female Fertility Promotion, Centre for Reproductive Medicine, Department of Obstetrics and Gynaecology, Peking University Third Hospital, Beijing, China; Department of Obstetrics and Gynaecology, School of Clinical Sciences, Monash University, Melbourne, VIC, Australia; National Clinical Research Centre for Obstetrical and Gynaecological Diseases, State Key Laboratory of Female Fertility Promotion, Centre for Reproductive Medicine, Department of Obstetrics and Gynaecology, Peking University Third Hospital, Beijing, China; National Clinical Research Centre for Obstetrical and Gynaecological Diseases, State Key Laboratory of Female Fertility Promotion, Centre for Reproductive Medicine, Department of Obstetrics and Gynaecology, Peking University Third Hospital, Beijing, China

Dear Editor,

We thank colleagues Esteves and Humaidan for their Letter (Esteves and Humaidan, 2026) regarding our article on sperm DNA fragmentation index (DFI) testing (Wang *et al*, 2026)—a secondary analysis including 953 of 2329 couples within our original randomized controlled trial (RCT) comparing ICSI versus IVF (Wang *et al.*, 2024). We appreciate their comments regarding selection bias, statistical power, interaction test, and overall interpretation in the context of clinical knowledge. While we already tried to address these points in our article (Wang *et al*, 2026), we provide brief responses to each of their comments below.

First, regarding selection bias, we have acknowledged that sperm DFI testing was not routinely performed across all sites, compromising randomization in the original trial. Consequently, this secondary analysis was treated as a cohort study rather than an RCT. Baseline characteristics of included couples were comparable, with the exception of endometrial thickness, which differed for continuous analysis but not for categorical analysis (Supplementary Table S3 in Wang *et al*., 2026). Therefore, subsequent modeling analyses adjusted only for center effects to address inconsistencies in treatment protocols across centers. As we have acknowledged, this analytical approach does not change the cohort classification of this secondary analysis, so the findings do not provide Level 1 evidence from an RCT.

Second, concerning the statistical power in subgroups with very high DFI levels (e.g. >40%), as our study population were couples with non-severe male infertility, it is reasonable that most participants did not have markedly high DFI values. The number of patients with DFI >40% was approximately 20 in each group, which made further subgroup analyses challenging. In fact, our results show that the ICSI group in Q4 (DFI ≥ 25.9%) had a lower rate of total fertilization failure than IVF, though with very few events (fewer than 10 per group, likely by chance). We made a cautious recommendation that DFI ≥ 25% might favor ICSI to avoid repeated fertilization failure, but this hypothesis requires further validation in well-designed, adequately powered RCTs.

In response to the comment by Esteves and Humaidan (2026) on the choice of statistical methods, the exploration of non-linear interactions, including multivariable fractional polynomial interaction analyses (that we used), and other approaches such as restricted cubic splines (Pham *et al*, 2025) are recommended approaches to address such clinical questions (Lopez-Ayala *et al*, 2025). In contrast, the dichotomization approach mentioned by Esteves and Humaidan (2026) introduces information loss (Lopez-Ayala *et al*, 2025). We thank Esteves and Humaidan (2026) for mentioning ‘Marker Sequential Test (MaST) Design’ (Freidlin *et al*, 2014), and we would be interested in collaborating with them to apply such approaches in future similar studies.

We agree with Esteves and Humaidan’s other comments regarding careful interpretation in the context of clinical variations (Esteves and Humaidan, 2026). There is ongoing debate about the value of sperm DNA fragmentation in ART decision-making. Given the heterogeneity of study populations, testing methods, and threshold values across different studies, these debates may be further amplified. More importantly, we should advocate for future research to deepen the understanding of sperm DNA damage mechanisms, develop more reliable and stable testing methods, and conduct well-designed clinical research across diverse patient subgroups to generate high-quality evidence that clarifies the value of sperm DNA fragmentation for precise diagnosis and treatment of infertility.
